# Knowledge, Attitude and Practice about Vitamin D among Pregnant Women at a Municipality of Bhaktapur

**DOI:** 10.31729/jnma.5496

**Published:** 2020-12-31

**Authors:** Pratibha Manandhar, Naresh Manandhar, Sunil Kumar Joshi

**Affiliations:** 1 Department of Community Medicine, Kathmandu Medical College, Duwakot, Bhaktapur, Nepal

**Keywords:** *attitude*, *knowledge*, *practice*, *qualitative study*, *vitamin D*

## Abstract

**Introduction:**

Almost a billion people in the world are affected by Vitamin D deficiency. During pregnancy, the deficiency of Vitamin D can manifest as gestational diabetes, pre-eclampsia, preterm birth, or miscarriage in the early stages of pregnancy. The objective of this study was to assess the knowledge, attitude and practice and importance of Vitamin D among pregnant women via focus group.

**Methods:**

A qualitative study was carried out at Changu Narayan Municipality, Duwakot ward no. two, Bhaktapur district from November to December 2019 after approval from Institutional Review Committee of Kathmandu Medical College (Ref. 181020192). Participants were selected via the Female Community Health Volunteer's pregnant women list by purposive sampling method. Focus group discussion was conducted among pregnant women. The interview questions were open-ended and the data were analyzed using thematic analysis.

**Results:**

Study participants showed limited knowledge on vitamin D. Few participants had information regarding sun exposure for vitamin D. But many participants had negative attitudes towards sun exposure and lack of knowledge on sun exposure requirements. The participants have a huge knowledge gap between Vitamin D and its importance in pregnancy.

**Conclusions:**

Increasing awareness of the importance of Vitamin D among pregnant women is required.

## INTRODUCTION

Vitamin D is a fat-soluble, sunshine hormone needed during infancy, adolescence, adulthood, and pregnancy. It is important for normal calcium absorption from the gut and bone growth.^1,2^ Cutaneous synthesis of Vitamin D is obtained by the conversion of 7-dehydrocholesterol to Cholecalciferol (Vitamin D3) by ultraviolet radiation from the sun.^3,4^

Studies reported a worldwide epidemic of 18-84% Vitamin D deficiency among pregnant women. Local clothing customs, limited exposure to sunlight, sunblock cream use, environment pollution, inadequate knowledge regarding Vitamin D might be the reasons.^5–10^ During pregnancy, Vitamin D deficiency can lead to pre-eclampsia, premature labour, miscarriage and gestational diabetes leading to increase risk of poor neonatal outcomes.^11,12^ Poor skeletal mineralization in-utero induced by maternal vitamin D deficiency may manifest as congenital rickets, craniotabes, and osteopenia in newborn infants.^13,14^

The objective of this study is to assess the knowledge, attitude and practice of Vitamin D among pregnant women at Duwakot in a tertiary care center.

## METHODS

A qualitative study in the form of focus group discussion (FGD) was conducted at Duwakot, Changu Narayan Nagarpalika, ward no. 2, Bhaktapur district, Nepal from November to December 2019. Ethical approval was obtained from the Institutional Review Committee (IRC) of Kathmandu Medical College (Ref.181020192). Participants were selected by the pregnant women list of local ward no. two Female Community Health Volunteer (FCHV). Participants were informed regarding the objective of the study and written informed consent was obtained maintaining their confidentiality. Purposive sampling method was applied. The interview questionnaires were open- ended and the data were analyzed using thematic analysis.

Rational behind choosing this FGD technique was participants were recruited from different ethnic caste groups and consist of primi and multigravida women maintaining the homogeneity of the group. For participant's comfort, confidence, and maintaining confidentiality, FGD was done at Kathmandu Medical College Hospital, Duwakot premises with the rapport building by local Female Community Health Volunteer (FCHV). The local FCHV was very familiar to them and there was enough space at the hospital premises and the hospital was familiar for their regular antenatal checkup. The recommended size of FGD may vary between four to six participants and duration of FGD depends upon saturation of data. In this study two FGDs was involved which consisted six pregnant women in each group in two setting. The duration of each FGD lasted for 25-35 min. Questionnaires were semi structured open ended adapted from the literature consisting of Vitamin D knowledge, its importance and sunlight exposure and audio recording were done with note-taking by the first author.^15,16,17^ Open ended questions regarding knowledge was about what do they understand about Vitamin D and its formulation. Similarly for practices, how often they stay in sunlight and do they wear full sleeved clothes or scarf or sun block while staying in Sunlight were asked. Open ended questions for attitude, whether they liked to exposed in Sun, More specific queries regarding personal sun exposure during the postnatal period were also asked for multigravida pregnant women. Recorded interviews were fully transcribed in the Nepali Language after listening and were translated into English. Transcription and translation were reviewed. The data was manually done. The thematic analysis was done. In the thematic analysis, data was manually identified by codes and themes. The coding process and theme development were accomplished. All the codes were rechecked and modified accordingly and extracted the common themes based on issues raised by the participants. The authors discussed and verified all the developed themes. The observational biases were tried to minimize.

## RESULTS

A total of 12 pregnant women were enrolled in this FGDs. The saturation of the data was determined at the point as no more new issues were raised. Table 1 shows the socio-demographic characteristics of the study participants (Table 1).

**Table 1:** Socio-demographic status of pregnant women

Variables	n (%)
**Age**	
< 20 yrs	2 (16.7)
20 - 25 yrs	3 (25.0)
26 - 30 yrs	7 (58.3)
Total	12 (100)
**Gravida**	
Primi	6 (50.0)
Multi	5(41.6)
Grand Multi	1 (8.4)
Total	12 (100)
**Educational status**	
Illiterate	2 (16.6)
Primary school	3 (25.0)
Secondary school	3 (25.0)
Intermediate	4 (33.4)
Total	12 (100)
**Occupation**	
Homemaker	8 (66.6)
Job holder	1 (8.4)
Teacher	1 (8.4)
Business	2 (16.6)
Total	12 (100)
**Ethnicity**	
Brahmin	5(41.6)
Chhetri	1 (8.4)
Janjati	6 (50.0)
Total	12 (100)

During the data analysis, four broad themes were revealed: (1) Knowledge, (2) Attitude (3)Importance of vitamin D (4) Practice

Sub-themes of the board themes are: (1) Knowledge: sources, information and importance of Vitamin D (2) Attitudes: duration and discouragement of sunlight exposure (3) Importance of vitamin D: pregnant women and infants (4) Practices: sun exposure after delivery.

This sub-theme has been classified for identifying the details of knowledge, attitude, practice and importance of vitamin D.

**1. Knowledge, sources and importance of vitamin D**

Participants were asked regarding vitamin D and importance. Most of the participants had no idea regarding knowledge, source and importance of vitamin D.

[P7]: “No idea regarding Vitamin D [Laughs]”

[P2]: “Vitamin D is received from mushroom, food which we eat”

[P7]: “Vitamin D is received from green vegetables and soup made from beans

[P8]: “Vitamin D is available from peanuts and cashew nuts”

[P9]: “Vitamin D is found in ice creams too when we consume lots of ice creams it makes skin brighter.

[P10] “We will be free from diseases [after sun exposure]... might be.[laughs]. Sun exposure will be healthy for people suffering from jaundice. Haha. [All participants laugh

However, in response to these questions, only two participants mentioned the exact source of Vitamin D.

[P8]: “Vitamin D means our Nature provides ...[pause] from sunlight in the early morning”

[P2]: “Vitamin D is available from Sunlight.

Besides, two participants mentioned Vitamin D tablets as supplements.

[P1]: “Doctors prescribe medicines containing Vitamin D to us as we do not take enough food. So, it [Vitamin D] is available in medicines also.

[P3]: “Calcium tablets contain Vitamin D”.

**2. Attitude, duration and Sun exposure discouragement**

Most of the pregnant women's attitude towards sun exposure was to stay warm and spent a few hours in sunlight rather than the concept of receiving vitamin D. Some women even ignored sun exposure too. As data were collected in the winter season, some of the participants expressed dissatisfaction on Sun exposure during summer. Due to headache and dizziness, few participants avoided sun exposure.

[P11]: “I feel cold that's why I stay in sunlight. Sun gives warmth.”

[P4]: “I too don't like sun, Its too hot in Summer [Laughs]”

[P5]: “I don't want to expose in the sunlight”

[P10]: “Sun exposure makes me feel dizzy and I avoid sunlight as it causes headache.”

Most of the participants didn't know duration and period to be spent in the sunlight. Few participants were even confused regarding the required period and duration of sun exposure.

[P8]: “We stay in the sun as much as we want [laugh], we don't see the time.”

[P3]: “Till we feel hot and very warm then we will go inside the room[smiles].”

[P7]: “I think 2-3 hours is enough.”

Even though participants had no adequate information regarding sun exposure, few participants protected themselves from the sun by using sunblock cream, wearing a scarf over the forehead and long sleeve clothes while on sun exposure. These behaviours could prevent people from getting sufficient sun exposure.

[P3]: “While returning from office, I must use scarf over the forehead.”

[P11]: “I apply sunblock cream on my face while staying in the sun. But, I don't apply it over hands. I believe it should be applied over the face. But as it [Sunscreen cream] stinks, I don't apply regularly. [laughs]”

**3. Knowledge regarding the importance of vitamin D for pregnant women and infants**

Almost all participants didn't have adequate knowledge about the importance of vitamin D for pregnant women

[P1]: “I don't have any idea”.

[P6]: “ I don't know.”

The reason behind sun exposure to infants was lacking among the participants who were primigravida as compared to multi. Even though one participant saw infants exposed in sunlight for oil massaging and one participant had an experience of keeping her baby in sunlight during postnatal period. Even though one participant mentioned health benefits like sun exposure reduces jaundice in neonates, makes baby stronger. But, they did not know that these benefits are related to Vitamin D received from sunlight have beneficial effects.

[P4]: “ Maybe it reduces jaundice in newborn babies, but I am not sure...[confused]”

[P4]: “Baby could catch from pneumonia, if not heated in sunlight... [pause].”

[P1]: “It will make babies' hand and leg bones stronger” One participant shared her different experience regarding the use of phototherapy to her baby at the hospital due to jaundice.

[P11]: “My previous baby developed jaundice at birth. So, the doctor kept my baby under the photo-therapy in the warm cot rather than keeping in sunlight. I had to stay in the hospital until my baby turned one month.”

**4. Practices regarding sun exposure after delivery**

A developing country Nepal has its cultural diversity. After delivery, every postpartum woman along with infants are exposed in sunlight from the ancient period. Even though they have seen mothers and babies kept in sunlight for oil massage, they didn't know the reasons and health benefits behind it.

[P4]: “We do oil massage for baby in sunlight[Smiles]. Especially Grandmothers do so for their grandchildren keeping the baby naked in sunlight and dressed up after that.”

[P2]: “We don't expose our face in the sunlight. We expose our back and then massage oil in sunlight and do the same for infants too. Some might stay aside and do massage, but I don't like to stay in sunlight for a long period.”

After the author's explanation regarding the reason behind Sun exposure is to receive vitamin D from the sun. Then the participant's reacted

[P3]: “It's our culture to keep babies in sunlight. We massage both mother and baby and stayfor a few hours in the sun. But, before this meeting, we didn't know that Vitamin D is available through sunlight.”

[P2]: “We do have our culture of this [Sunlight exposure of mother and baby with oil message] since the ancient day, but didn't know the reason behind it. It's really for vitamin D! Ohhh.. [Exclamatory reaction]. Now, we knew it. [smiles].”

## DISCUSSION

This qualitative approach was used to explore knowledge and attitudes regarding vitamin D among pregnant women in Duwakot. This study scrutinized social and cultural factors that potentially contribute to prevent vitamin D deficiency among postnatal mothers and their newborns by sunlight exposure until postnatal 2 months. This study has revealed a key finding that participants had limited knowledge regarding vitamin D and its deficiency. Few participants knew to understand as Sun exposure is the main source of vitamin D and food sources of vitamin D as well. This information was received from literate participants.

A qualitative study done by Bonevski et al. in Sydney, Australia found limited knowledge of vitamin D among adults participants.^18^ They were not aware of Sun exposure duration and body parts to be exposed for vitamin D. In our study also, pregnant women showed similar ignorance towards Vitamin D. Furthermore, poor knowledge on dietary vitamin D source was also noticed. But, two participants mentioned the importance of vitamin D which helped them to understand betterment in solving the problems related to Vitamin D deficiency.

Our participants had negative attitudes toward sun exposure. Participants prefer to wear full sleeve dress, use Sun block cream, umbrella and scarf for Sun barrier. Health conditions like dizziness and headache were the reasons behind refusing sun exposure. A qualitative study by Webb AR et al. in Greater Manchester, UK also found above mentioned similar negative attitudes.^19^ In their study, participants were confused regarding sunlight exposure (risk/benefit) for vitamin D and the use of Sun block cream for preventing Sunburnt and skin cancer. These findings showed there is a need for health education to pregnant women regarding the importance of vitamin D.

One participant had information regarding the requirement of sun exposure to postnatal mother and baby. But, there is a lack of knowledge regarding its benefits and importance. One participant knew about the health benefits of sun exposure to babies like reducing jaundice, making babies stronger. A study done by Gedamu H et al in Ethiopia found that nearly half of the participants knew sunlight exposure to their infant but the reasons behind keeping babies in the sunlight were not mentioned.^20^ These findings were similar to our study also as most of the pregnant women were unknown regarding the importance of sun exposure to babies.

The interviews were conducted in local Nepali language allowing direct communication with the study participants to gain direct insight into the issues embedded in a complex social and cultural context, which helped participants to feel more trust and to speak up easily to share their experiences, which is the great strength of this study. A lot of quantitative studies on vitamin D were published worldwide, but in our country, there is a shortage of qualitative studies on knowledge, attitudes and practice (KAP) exploring social and cultural factors influencing vitamin D. This study has added further evidence to explore the underlying issues related to vitamin D and its deficiency among pregnant women. However, in this study, only two FGDs with twelve participants included, the findings cannot be generalized.

## CONCLUSIONS

Based on our findings, awareness of the importance of vitamin D among the different groups of the population via health education and various promotional activities are required. Furthermore, these activities should focus on the requirement of sun exposure duration, a period of the day and proportion of body parts to be exposed in the sun. It is very important to receive sufficient vitamin D apart from enjoying the warm sun in winter and not escaping from the sun during the summer. The knowledge about the long-term effect of vitamin D deficiency and its correlation with chronic diseases should also be explained to pregnant women and postpartum mothers.
